# Blood pressure control in type 2 diabetic patients

**DOI:** 10.1186/s12933-016-0485-3

**Published:** 2017-01-06

**Authors:** Alon Grossman, Ehud Grossman

**Affiliations:** 1 Department of Internal Medicine E, Rabin Medical Center, Petach Tikva, Israel; 2 Department of Internal Medicine D and Hypertension Unit, The Chaim Sheba Medical Center, 52621 Tel-Hashomer, Israel; 3 Sackler Faculty of Medicine, Tel-Aviv University, Tel-Aviv, Israel

**Keywords:** Hypertension, Blood pressure, Diabetes, Review

## Abstract

Diabetes mellitus (DM) and essential hypertension are common conditions that are frequently present together. Both are considered risk factors for cardiovascular disease and microvascular complications and therefore treatment of both conditions is essential. Many papers were published on blood pressure (BP) targets in diabetic patients, including several works published in the last 2 years. As a result, guidelines differ in their recommendations on BP targets in diabetic patients. The method by which to control hypertension, whether pharmacological or non-pharmacological, is also a matter of debate and has been extensively studied in the literature. In recent years, new medications were introduced for the treatment of DM, some of which also affect BP and the clinician treating hypertensive and diabetic patients should be familiar with these medications and their effect on BP. In this manuscript, we discuss the evidence supporting different BP targets in diabetics and review the various guidelines on this topic. In addition, we discuss the various options available for the treatment of hypertension in diabetics and the recommendations for a specific treatment over the other. Finally we briefly discuss the new diabetic drug classes and their influence on BP.

## Background

Essential hypertension and diabetes mellitus (DM) are both extremely common conditions and therefore it is not surprising that their co-existence is extremely prevalent. Since both are considered risk factors for coronary artery disease, cerebrovascular disease, renal failure and congestive heart failure, treatment of both conditions is essential. Whether blood pressure (BP) should be lowered to a different target in diabetic patients has been a debate for many years. Current guidelines are inconsistent regarding BP target in diabetic patients. Whereas several guidelines recommend a BP goal of <140/90 mmHg [1, 2], some recommend a lower target for diastolic BP [3, 4] and some recommend lower systolic BP thresholds in certain diabetic population [5–7]. The class of drug treatment most appropriate for the treatment of hypertensive diabetics is also unclear and different guidelines emphasize use of different drug classes for the treatment of hypertension in diabetic patients. Recently introduced drug classes for the treatment of DM have also been found to lower BP, thus making the interaction between BP and DM even more complex. In this review, we discuss the epidemiology of diabetes and hypertension, the benefit of lowering BP in diabetic patients, the target BP and the recommended treatment to achieve the target in these patients. This review deals mainly with BP control in type 2 DM, but some of the data derived from studies that included also non diabetic patients.

### Epidemiology

Hypertension is twice more common in diabetics than in non-diabetics [8], but the definition of hypertension in diabetics is generally similar to the general populations and the threshold for treatment is persistent BP values ≥140/90 mmHg. As both hypertension and DM are highly associated with obesity, it is not surprising that their co-existence is particularly common in obese individuals [9]. Both hypertension and DM increase significantly with increasing age and their co-existence is highest in older individuals [10]. Patients with DM more commonly present with isolated systolic hypertension and are more resistant to treatment. In the EUROASPIRE IV survey only 54% of the diabetic patients achieved BP levels of less than 140/90 mmHg [11]. In addition, the presence of autonomic neuropathy in diabetic patients is associated with a less nocturnal BP decrease, a higher baseline heart rate and a higher BP variability than in non-diabetics [12–18].

The co-existence of DM and hypertension significantly increase the risk for coronary heart disease [19], left ventricular hypertrophy [20], congestive heart failure [21] and stroke [22] compared with either condition alone. In addition, both hypertension and DM are present in all prediction models for the occurrence of stroke in patients with atrial fibrillation [23–25]. Microvascular complications are also more common in patients with co-existent hypertension and DM and both retinopathy and nephropathy are more prevalent in patients with DM and hypertension [26, 27]. Lowering BP is particularly beneficial in diabetic patients [28, 29], however how low should BP be is controversial.

### What should be the blood pressure target in diabetes mellitus?

The BP targets in diabetic hypertensive individuals are controversial. For many years it was common practice to aim for BP targets lower than 130/80 mmHg in non-proteinuric diabetic patients. This was based on evidence from several large studies, including The Hypertension Optimal Treatment (HOT) study, the United Kingdom Prospective Diabetes Study (UKPDS) 38 and the Action in Diabetes and Vascular disease Controlled Evaluation (ADVANCE) trial [29–31]. However, in most studies the achieved BP was higher than 135/85 mmHg and therefore the recommendation to lower BP to less than 130/80 mmHg was not solid [32, 33]. Moreover, several studies reported no benefit and even harm when lower BP targets were achieved. In the Ongoing Telmisartan Alone and in Combination with Ramipril Global End point Trial (ONTARGET) study, which included 9612 diabetic patients, the composite primary outcome of death from cardiovascular (CV) causes, myocardial infarction, stroke, or hospitalization for heart failure did not differ between groups despite achievement of lower BP values in the telmisartan-ramipril arm [34]. In the Prevention Regimen for Effectively Avoiding Second Strokes (PROFESS) trial, which included 5743 diabetics, recurrence of stroke was not less in patients receiving telmisartan despite a significant decrease in BP [35]. The PROFESS results were different from those of the Perindopril Protection Against Recurrent Stroke Study Collaborative Group (PROGRESS) trial [36], in which treatment with the ACE inhibitor perindopril was associated with a 38% risk reduction in the occurrence of stroke, but the PROGRESS trial included only 762 diabetic patients and they were recruited much longer following the initial stroke than in the PROFESS trial. The Telmisartan Randomised Assessment Study in ACE Intolerant Subjects with CV Disease (TRANSCEND) study [37] was another study which 35.7% of the patients were diabetics and in which more significant BP reduction with telmisartan was not associated with CV benefit.

In the International Verapamil SR/Trandolapril (INVEST DM) study there was no difference in short term outcome in diabetic patients with coronary artery disease despite achievement of significantly lower BP (<130 mmHg vs. <140 mmHg) and in fact, there was an increased in the long term all-cause mortality in the more tightly controlled group [38]. In the Action to Control Cardiovascular Risk in Diabetes Blood Pressure (ACCORD-BP) trial, BP reduction to <120 mmHg did not reduce mortality or overall CV outcomes, but did reduce significantly only the occurrence of stroke a pre specified secondary outcome [39]. Moreover, intensive BP lowering was associated with an increased rate of syncope and hyperkalemia, both directly related to the intensive treatment. The results of this large prospective study, in addition to data from other studies, led most of the societies to recommend less stringent BP target in diabetic patients.

However, the results of the recent systolic blood pressure intervention trial (SPRINT) raised again the discussion what should be the target BP in diabetic patients. The SPRINT randomized 9361 persons with systolic BP > 130 mmHg and increased CV risk, but without type 2 DM, to a systolic BP target <120 mmHg (intensive treatment) or a target of <140 mmHg (standard treatment). At 1 year, the mean systolic BP was 121.4 mmHg in the intensive treatment group and 136.2 mmHg in the standard-treatment group. The study was stopped early after a median follow-up of 3.26 years owing to 25% lower rate of the primary composite outcome in the intensive-treatment group than in the standard-treatment group (P < 0.001). All-cause mortality was also lower by 27% in the intensive treatment group (P = 0.003) [40]. The main benefit was observed in elderly subjects (>75 years) who constituted 28% of the study population [41]. Rates of serious adverse events of hypotension, syncope, electrolyte abnormalities, and acute kidney injury or failure, but not of injurious falls, were higher in the intensive-treatment group than in the standard-treatment group [40]. This recent study supports intensive BP lowering in non-diabetic patients with increased CV risk. The most important question came from the SPRINT is related to diabetic patients who were excluded from this study. In light of the discrepancy between the ACCORD and the SPRINT can we assume that the better results in SPRINT with intensive BP lowering does not apply to diabetic patients?

One approach is to explain why the results of the SPRINT should not be applied to diabetic patients and, unlike our previous thoughts BP targets in diabetic patients should be higher than in non-diabetics. DM has a negative influence on arteriolar function and blood flow autoregulation that shifts the pressure/flow relationship. Therefore diabetic patients are more vulnerable to compromised blood flow to vital organs when BP reaches a critical low point.

The opposite approach is that the results of the SPRINT should be applied to diabetic patients, since in most previous trials the benefits of BP reduction in diabetic patients were at least as good if not better than in non-diabetic individuals [29, 42]. To justify this approach one should look at the effect of intensive BP lowering in diabetic patients on stroke, the long-term follow up results of the ACCORD study and the differences between the ACCORD and the SPRINT.

In the ACCORD study, despite the failure to show a decrease in primary endpoints in the intensive treatment arm the rate of stroke was significantly lower in the intensive than in the usual treatment arm [39]. It is possible that the ACCORD trial was underpowered, with a much lower event rate than anticipated and therefore the benefit of intensive BP lowering was not observed. Recently, new results from a long-term follow-up of the ACCORD patients, dubbed the ACCORDION trial, were presented at the 2015 AHA meeting [43]. In this extended study 3957 patients were followed for an additional 54–60 months. During this time, patients who had been in the intensive BP arm in the main trial were no longer aiming for the lower BP goals, so the difference in BP between the two groups narrowed from 14.5 mmHg at the end of the main trial to 4.2 mmHg at the end of the follow-up period. Results from the follow-up period showed a 9% non-significant reduction in the primary end point of major CV events over a median follow-up of 8.8 years from randomization. During the long-term follow-up, an interaction between BP and glycemia interventions became significant (P for interaction 0.037), with evidence of benefit for intensive BP lowering in participants randomized to standard glycemia therapy (HR = 0.79, 95% CI 0.65–0.96). These long-term results of the ACCORD trial do take on enhanced importance when viewed alongside the SPRINT results.

Several differences in the design of the studies may also explain the different results. ACCORD had lower event rates than initially predicted because of a lower CV risk profile in participants. The exclusion of participants aged >80 years led to a younger group of patients in ACCORD than in SPRINT. The mean age for ACCORD was 62 years and for SPRINT was 68 years.

Participants in the BP arm of the ACCORD were also at lower risk because patients with dyslipidemia were assigned to the lipid arm and were excluded from the BP arm.

Another significant difference in the design of the SPRINT and ACCORD studies was the use of diuretics. The treatment regimen for hypertension in the ACCORD study often used hydrochlorthiazide, and the SPRINT study primarily used chlorthalidone.

In addition, the complexity of the factorial study design in ACCORD may have made it less likely that a statistically significant difference could be demonstrated. This may suggest that if diabetic patients were included in the SPRINT they would also benefit from intensive BP lowering.

When we try to explain the reason for the difference between the SPRINT and the ACCORD it should be emphasized that the results of the SPRINT are provocative. In the recent Heart Outcomes Prevention Evaluation (HOPE)–3 trial 12,705 participants at intermediate risk who did not have CV disease were randomized to receive either candesartan at a dose of 16 mg per day plus hydrochlorothiazide at a dose of 12.5 mg per day or placebo and were followed for 5.6 years. The first co-primary outcome was the composite of death from CV causes, nonfatal myocardial infarction, or nonfatal stroke; the second co-primary outcome additionally included resuscitated cardiac arrest, heart failure, and revascularization. Therapy with candesartan plus hydrochlorothiazide was not associated with a lower rate of major CV events than placebo despite a BP decrease of 6.0/3.0 mmHg in the active treatment group. The only subgroup who benefited from BP lowering was the subgroup of participants with initial systolic BP > 143.5 mmHg [44]. A recent study that used the extended follow-up data from the US cohort of the International Verapamil [SR]/Trandolapril Study (INVEST) showed that in hypertensive patients with coronary artery disease, achieving a systolic BP of 130–140 mmHg seems to be associated with lower all-cause mortality after approximately 11.6 years of follow-up [45]. Similarly, the Secondary Prevention of Small Subcortical Strokes (SPS3) trial) evaluated BP goals in patients with a previous lacunar stroke testing a systolic goal of 130–149 mmHg versus <130 mmHg [46]. This trial also did not demonstrate significant reductions in ischemic stroke or intracranial hemorrhage in the more intensive treated group. Why the results of the SPRINT showed a clear benefit of lowering systolic BP to <120 mmHg whereas other studies failed to show it?

One explanation is the technique of BP measurements. In the SPRINT, BP was measures with an automated oscillometric office BP method that eliminated the need for a human to participate in the actual measurement and therefore reduces the white coat effect. Compared with a reasonably well-done standard office-based BP, the use of an automated oscillometric office BP method will yield a systolic BP that is 7–10 mmHg lower in the same patient, measured on the same day. If this is true the systolic BP of 120 mmHg in the SPRINT is equivalent to almost 130 mmHg in clinical practice. Thus, it is reasonable to suggest in high risk patients a target systolic BP of <130 rather than <120 mmHg.

To solve the discrepancy between the various studies and to find out what should be the target systolic BP several meta- analysis were recently published (Table 1).

**Table 1:** Meta-analyses of anti-hypertensive treatment in diabetic patients

Topic	Year	Journal	Number of studies included	Number of patients included	Number of diabetics	Mean follow-up (years)	Main conclusions
Effect of antihypertensive treatment at different BP levels in patients with diabetes mellitus [47]	2016	British Medical Journal	49	73,738	Only diabetic, most type 2	3.7	If BP was greater than 150 mmHg, treatment reduced all-cause mortality, CV mortality, myocardial infarction, stroke and end stage renal disease. If baseline systolic BP was less than 140 mmHg, further treatment increased the risk of CV mortality with a tendency towards an increased risk of all-cause mortality
BP lowering for prevention of CV disease and death [49]	2016	The Lancet	123	613,815	NA	NA	Every 10 mmHg reduction in systolic BP significantly reduced the risk of major CV disease events, coronary heart disease, stroke and heart failure which, in the populations studied, led to a significant 13% reduction in all-cause mortality. The effect on renal failure was not significant. Proportional risk reductions (per 10 mmHg lower systolic BP) were noted in trials with higher mean baseline systolic BP and trials with lower mean baseline systolic BP. There was no clear evidence that proportional risk reductions in major CV disease differed by baseline disease history, except for diabetes and chronic kidney disease, for which smaller, but significant, risk reductions were detected
BP targets for hypertension in people with diabetes mellitus [48]	2013	Cochrane Database systematic reviews	5	7314	7134	4.5	Reduction in incidence of stroke in intensive BP reduction compared with standard reduction, no effect on mortality, significant increase in other serious adverse events
BP Targets in Subjects With Type 2 Diabetes Mellitus/Impaired Fasting Glucose [50]	2011	Circulation	13	37,736	All	4.8 ± 1.3	A systolic BP treatment goal of 130 to 135 mmHg is acceptable. However, with more aggressive goals (<130 mmHg), the risk of stroke continues to fall, but there is no benefit regarding the risk of other macrovascular or microvascular events, and the risk of serious adverse events even increased
Effects of intensive BP reduction on myocardial infarction and stroke in diabetes [51]	2011	Journal of Hypertension	31	73,913	159	NA	Tighter BP control reduced the risk of stroke by 31% compared with less tight control, whereas the reduction in the risk of MI was not significant

CV, cardiovascular; BP, blood pressure, NA, not available; MI, myocardial infarction

A meta-analysis of 49 trials including 73,738 patients (most of them diabetic) showed that at BP values greater than 140 mmHg, BP reduction was associated with a decrease in mortality and CV morbidity. On the other hand, BP reduction in patients with initial BP values <140 mmHg resulted in increased CV mortality and a tendency towards increased overall mortality [47]. Another meta-analysis evaluated randomized controlled trails performed only in diabetic individuals and concluded that the present evidence does not support BP targets lower than the standard targets in people with elevated BP and diabetes [48]. A recently published meta-analysis evaluated BP lowering for prevention of CV disease and death and reported that the proportional reduction in major CV disease events by BP reduction seemed to be larger in trials done in people without diabetes or chronic kidney disease [49]. This was attributed to different methodological characteristics in studies in diabetic patients. Another meta-analysis of 13 randomized control studies including over 37,000 diabetic hypertensive patients has shown that intensive systolic BP control to less than 130 mmHg was associated with a 10% reduction in all-cause mortality, yet no effects on microvascular or macrovascular events were noted. Regarding stroke, such an intensive BP reduction has led to a 17% risk reduction, accompanied by an additional risk reduction with further lowering systolic BP to <120 mmHg, without an increased risk for adverse effects [50]. Another meta-analysis included 31 randomized control studies with over 73,000 diabetic hypertensive patients reported a 31% reduction in relative risk of stroke, with a 13% reduction for every 5 mmHg systolic BP or 2 mmHg diastolic BP reductions. The risk of myocardial infarction was not significantly reduced with a more intensive BP control [51].

Thus it seems that a target of systolic BP < 130 mmHg is reasonable in most diabetic patients. In elderly diabetic patients (>80 years) but otherwise healthy, a BP target of <140–150/90 mmHg is reasonable. Lower BP levels may be adequate if tolerated by the patients. BP levels should be monitored closely in the sitting and the standing position and the treatment should be tailored to prevent excessive fall in BP [52].

### Treatment goals according to current guidelines

Although previous guidelines recommended strict BP control in diabetic patients [53, 54], this has been challenged in recent guidelines (Table 2). The British National Institute for Health and Clinical Excellence (NICE) guidelines published in 2011 [55] recommended commencing treatment in diabetic patients with stage 1 hypertension (Clinic BP > 140/90 mmHg and ambulatory BP monitoring (ABPM) daytime average or home BP monitoring (HBPM) average BP of >135/85 mmHg). The recently published 2016 American Diabetes Association (ADA) guidelines recommended that hypertensive diabetic patients be treated if they have a diastolic BP of >80 mmHg or a systolic BP > 140 mmHg, with a target BP value of <140/90 mmHg [6]. These guidelines state that individuals in whom stroke risk is a concern may, as part of shared decision making, have lower systolic targets such as 130 mmHg. This is especially true if lower BP can be achieved with few drugs and without side effects of therapy. The American Heart association (AHA)/American College of Cardiology (ACC) guidelines from 2014 recommend a target BP of <140/90 mmHg, but point out that lower targets may be considered [56]. The American Society of Hypertension (ASH)/International Society of Hypertension (ISH) guidelines from 2014 suggest a BP goal of <140/90 mmHg in diabetic patients [2]. These values are lower than those recommended by the majority of the JNC 8 panel for non-diabetic patients aged 60–79, which was <150/90 mmHg, yet similar to those recommended for non-diabetics aged 18–60 years, and similar to the values of all non-diabetic patients by the minority view of the JNC8 [1]. The 2013 European Society of Hypertension (ESH) and European Society of Cardiology (ESC) guidelines recommend lowering systolic BP below 140 mmHg, and diastolic BP below 85 mmHg [3]. The Canadian Hypertension Education Program (CHEP) suggests a target BP of <130/80 mmHg [7]. The International Diabetes Federation (IDF) suggests age-adjusted BP targets (BP target values of <130/80 mmHg for diabetic patients younger than 70 years, target values of <140/90 mmHg for patients 70–80 years old, and target values of <150/90 mmHg for patients over 80 years old) [5].

**Table 2:** BP goals in diabetics according to major guidelines

Guidelines	NICE [54]	ESH/ESC [3, 4]	ASH/ISH [2]	JNC 8 [1]	ADA [6]	CHEP [7]	IDF [5]
Year published	2011	2013	2014	2014	2016	2016	2012
Blood pressure (mmHg)	Not addressed	<140/85	<140/90	<140/90	<140/90	<130/80	<130/80
Special considerations	Begin treatment if BP > 140/90 mmHg				Systolic BP < 130 mmHg and diastolic BP < 80 may be appropriate for certain individuals with diabetes, such as younger patients, those with albuminuria, and/or those with hypertension and one or more additional atherosclerotic CV disease risk factors, if they can be achieved without undue treatment burden.		<140/90 mmHg in patients 70-80 years old <150/90 mmHg in patients over 80 years old
Recommended initial treatment	ACE inhibitor plus either a diuretic or a CCB	All classes of antihypertensive agents are recommended. RAAS blockers may be preferred, especially in the presence of proteinuria or microalbuminua	ARB or ACE inhibitor. In black patients, it is acceptable to start with a CCB or a thiazide.	Thiazide-type diuretic, CCB, ACE inhibitor or ARB	ACE inhibitor, ARB	ACE inhibitor, ARB in patients with CV or kidney disease, including microalbuminuria, or with CV risk factors	In patients without albuminuria, Thiazide-type diuretic, CCB, ACE inhibitor or ARB

NICE, National Institute for Health and Clinical Excellence; ESH/ESC, European Society of Hypertension/European Society of Cardiology; JNC, Joint National Committee; ASH+, American Society of Hypertension; ISH, International Society of Hypertension; ADA, American Diabetes Association; CHEP, Canadian Hypertension Education Program; BP, blood pressure; ACE, angiotensin converting enzyme; CCB, calcium channel blocker; RAS, renin angiotensin system; ARB, angiotensin receptor blocker; BB, beta blocker

### How to reach goal blood pressure in diabetics

#### Non-pharmacological treatment

Non-pharmacological anti-hypertensive therapy includes weight loss, increased potassium-based diet (DASH- dietary Approach to Stop Hypertension- style diet), low sodium consumption (below 2400 mg/day), moderation of alcohol intake and regular physical activity and exercise. Although the CV benefits of lifestyle interventions were not evaluated in diabetic patients, their implementation seems reasonable in diabetics since they may positively affect glycemia and lipid profile. Therefore their adoption for all diabetic patients with BP values >120/80 mmHg was recommended by recent ADA standards of care [6].

### Pharmacological treatment

#### Renin-angiotensin-aldosterone blockers

Angiotensin converting enzyme inhibitors (ACEI), and angiotensin receptor blockers (ARBs) have long been considered the cornerstone of anti-hypertensive treatment in diabetic patients. Previous studies have demonstrated that both renin-angiotensin-aldosterone system (RAAS) blockers, ACEI and ARB, are associated with prevention of new onset DM in hypertensive patients [57] and are particularly favorable among patients with albuminuria [57]. Although ACEIs were reported to reduce overall CV risk, overt nephropathy, renal failure and retinopathy among non-hypertensive diabetics, other studies failed to show the superiority of ACEI over beta blockers in lowering BP and preventing nephropathy or retinopathy in diabetic patients [58, 59]. Despite the fact that ACEIs were found to be superior to ARBS in preventing all-cause mortality and CV morbidity and mortality in two meta-analysis [60, 61], in the ONTARGET study, outcome was similar between the two drug classes [34] and in a recent real-world study ARBS were found to be more effective than ACEI in the prevention of stroke [62]. Therefore it seems that ACEIs and ARBs are probably equally efficacious for the prevention of CV outcomes in hypertensive diabetics. ARBs and ACEIs are equally effective in preventing progression of kidney disease in diabetic patients with early nephropathy with ARBS having comparable BP lowering capacity with fewer side effects compared with ACEIs [63]. In a recent study that compared the BP lowering effect of ARBs in diabetic patients, azilsartan medoxomil was more effective than olmesartan and valsartan [64]. A recent meta-analysis of 19 randomized controlled trials with over 25,000 participants found that ACEIs or ARBs were associated with a similar risk of death (relative risk 0.99, 95% CI 0.93–1.05), CV death (1.02, 0.83–1.24), myocardial infarction (0.87, 0.64–1.18), angina pectoris (0.80, 0.58–1.11), stroke (1.04, 0.92–1.17), heart failure (0.90, 0.76–1.07), revascularization (0.97, 0.77–1.22) and end stage renal disease (0.99, 0.78–1.28) as compared with other anti-hypertensive agents [65]. Combining two RAAS blockers is discouraged based on the discouraging results of the Aliskiren Trial in Type 2 Diabetes Using Cardiorenal Endpoints (ALTITUDE) and the ONTARGET trials [34, 66]. In summary, it seems that use of ACEIs or ARBs is not superior to use of other anti-hypertensive agents in diabetics without evidence of nephropathy, but these classes are legitimate first-line treatment options in the absence of contraindications.

#### Beta blockers

The use of beta blockers has been discouraged in diabetic patients due to its potential adverse metabolic effects, including an increase in triglyceride levels, a decrease in HDL cholesterol levels, weight gain, masking hypoglycemia and impairing insulin sensitivity [67]. In addition, it has been suggested that use of beta blockers in non-diabetic individuals, particularly those who are overweight or obese, might increase the risk for development of diabetes compared with an alternative agent [68]. As beta blockers are being used infrequently as first-line agents for the treatment of hypertension, their use in diabetes is also infrequent, but beta blockers may still be used as add-on treatment in those who require multiple agents and in patients in whom another indication for the use of beta blockers is present, such as those with tachycardia, heart failure or ischemic heart disease [1, 3].

#### Calcium channel blockers (CCBs)

CCBs are considered a potential first-line treatment for hypertensive diabetics, particularly in the elderly with isolated systolic hypertension [69]. CCBs have been shown to be particularly effective in the prevention of stroke, but are less effective than RAAS blockers in prevention of heart failure [70]. Although non-dihydropyridines decrease urinary protein excretion and serve as an alternative in RAAS inhibitor-intolerant patients [71], most research in recent years has focused on the efficacy and safety of dihydropyridines. The Anglo-Scandinavian Cardiac Outcomes Trial (ASCOT BPLA) compared use of atenolol with amlodipine and found that amlodipine was more effective than atenolol in reducing stroke, CV events and all-cause mortality [67]. This advantage of amlodipine was evident in the large group of 5137 diabetics included in the study [72]. Notably, an ACEI was added to the amlodipine arm when BP was not controlled, whereas in the atenolol arm, a thiazide was added. A systematic review from 2015 evaluated the efficacy of amlodipine in the treatment of patients with hypertension with concomitant DM and/or renal dysfunction compared with other classes of antihypertensive medication and found that amlodipine was at least as effective as other anti-hypertensive agents in the treatment of hypertension, was associated with a decrease in stroke risk and an increase in heart failure risk [73]. CCBs are ineffective for the prevention of diabetes in non-diabetic individuals [74]. In summary, CCBs may be used as first-line agents for the treatment of hypertension in diabetic individuals, particularly in the elderly with isolated systolic hypertension.

#### Diuretics

Although there has been concern that diuretics might increase the risk for the development of diabetes mellitus [75] due to their potential to negatively influence insulin resistance [76], diuretics are important agents used for the treatment of hypertension in diabetics. In a sub-analysis of the Antihypertensive and Lipid-Lowering Treatment to Prevent Heart Attack Trial (ALLHAT), chlortalidone was found to be as good as amlodipine or lisinopril in preventing fatal and non-fatal coronary artery disease and was more effective in the prevention of heart failure in diabetic patients [77]. The benefits of diuretics were also observed in the SHEP trial [78, 79]. In all studies in which diuretics were found to be effective in hypertensive diabetics, chlorthalidone or indapamide were used. To summarize, diuretics may be used for the treatment of hypertension in diabetics either as first line agents or as add-on treatment, but glucose and electrolytes should be monitored when initiating therapy.

#### Alpha blockers

There are no specific studies which evaluated the efficacy of alpha blockers in diabetic patients. Alpha blockers do not adversely affect glucose metabolism or lipid profile, but they have been reported to be less effective than chlorthalidone for prevention of stroke and heart failure [80, 81] and therefore are used almost exclusively in patients with hypertension and prostate hyperplasia or as third or fourth-line agents.

#### Aldosterone antagonists

Low dose spironolactone was found to be effective in controlling BP in patients with hypertension and diabetes [82]. The addition of spironolactone is particularly effective in those with serum potassium of <4.5 mmol/L [83]. To prevent hyperkalemia thiazide or thiazide like diuretics should be continued when aldosterone antagonists are added [84]. The addition of spironolactone to conventional antihypertensive treatment in diabetic patients was shown to reduce albuminuria [85] and in diabetic patients with albuminuria, addition of an aldosterone antagonist to an ACEI has been shown to have renoprotective effects superior to those shown with the addition of an ARB, even when BP reduction rates were similar [86]. Finerenone is a new non-steroidal anti mineralocorticoid which has less relative affinity than spironolactone and eplerenone to other steroid hormone receptors, and therefore has less adverse effects like gynaecomastia, impotence, low sex drive and hyperkalemia. A recent study showed that in patients with diabetic nephropathy the addition of finerenone to an angiotensin-converting enzyme inhibitor or an angiotensin receptor blocker improved urinary albumin-creatinine ratio better than placebo [87]. It seems that aldosterone antagonists have a renoprotective effect that is independent of systemic hemodynamic alterations [88]. Diabetic individuals tend to develop type 4 renal tubular acidosis and therefore hyperkalemia may be a concern in those treated with aldosterone antagonists, particularly when combined with ACEIs or ARBs, although the long-term risk is low [89].

#### Combination therapy

More than two-thirds of hypertensive individuals are inadequately controlled on mono therapy [90]. Most diabetic individuals are treated with RAAS inhibitors and most guidelines recommend adding a calcium antagonist or diuretic as add-on therapy [1, 5, 6]. In a sub-analysis of 6946 diabetic patients, in the Avoiding cardiovascular Events through combination Therapy in Patients Living with Systolic Hypertension (ACCOMPLISH) trial, a combination of benazepril plus amlodipine was significantly more effective in reducing the composite of CV death, nonfatal myocardial infarction, nonfatal stroke, hospitalization for angina, resuscitation after sudden cardiac arrest, and coronary revascularization, compared to therapy with benazepril plus hydrochlorothiazide [42]. The superiority of amlodipine over hydrochlorothiazide as an addition to benazepril disappeared in obese individuals [91]. Combining a RAAS blocker with a CCB provides better renoprotection and leads to less ankle edema compared with a CCB alone [92]. In addition, combining an ARB with a CCB was associated with improved insulin sensitivity compared with an ARB and a diuretic [93]. Based on these studies, it seems that CCBs are appropriate as second-line agents in diabetic patients already treated with RAAS blockers. In obese individuals or when volume overload is present, diuretics may be used as well. In a large group of patients with stage I hypertension a combination of chlorthalidone and amiloride yielded a greater reduction in BP than the ARB losartan [94]. In patients requiring triple therapy, RAAS blockers should be combined with diuretics and CCBs, unless there is compelling indication for the use for a different anti-hypertensive class (heart failure or ischemic heart disease for beta blockers or benign prostate hyperplasia for alpha blockers). Patients with resistant hypertension, particularly in the presence of low potassium levels, may benefit from aldosterone antagonists. These should be used cautiously, particularly in patients already on RAAS blockers. Once BP goal has been achieved antihypertensive treatment should be continued. In the ADVANCE trial discontinuation of antihypertensive medications was associated with increased risk of combined macro and microvascular events [95].

### Diabetic treatment effective for the control of hypertension

In the last decade there is a surge of new anti-diabetic medications working on different pathways in insulin production and glucose disposal. Some of these agents have beneficial effects on BP and may prove as important agents for the control of hypertension in diabetic individuals. In this paragraph we will discuss these classes of agents and the evidence for their effect on elevated BP in both normotensive and hypertensive individuals.

#### Glucagon-like-polypeptide 1 analogues

Glucagon-like-polypeptide 1 analogues (GLP1a) lead to a clinically significant weight loss in both diabetics and non-diabetics [96, 97] and thus may aid in a better BP control. On the other hand, they have been reported to increase heart rate through sympathetic nervous system activation [98] and this may result in BP elevation. In the recently published Liraglutide Effect and Action in Diabetes: Evaluation of Cardiovascular Outcome Results (LEADER) trial [99], patients treated with liraglutide had a mild decrease in systolic BP (1.5 mmHg) and a mild increase in diastolic BP (0.6 mmHg). In the trial to evaluate CV and other long-term outcomes with Semaglutide in Subjects with Type 2 Diabetes (SUSTAIN-6) in which the CV safety of semaglutide was evaluated [100], the mean systolic BP in the semaglutide group, as compared with the placebo group, was 1.3 mmHg lower in the group receiving 0.5 mg (P = 0.10) and 2.6 mmHg lower in the group receiving 1.0 mg (P < 0.05). Thus it seems the GLP1a have a neutral effect on BP and may even result in a mild decrease in BP, but probably cannot serve as an alternative to anti-hypertensive treatment in hypertensive diabetics.

#### Dipeptidyl peptidase-4 (DPP4) inhibitors

DPP4 inhibitors elevate endogenous GLP1 through inhibition of the endogenous substance responsible for its degradation. Several studies reported that these agents produce a modest decrease in BP [101–103], others reported that they increase BP [104] and yet others reported that they negate the hypotensive effects of ACEI [105]. Overall it seems that DPP4 inhibitors are neutral in term of BP control and their initiation probably does not significantly affect BP control.

#### Sodium-glucose- transporter 2 (SGLT2) inhibitors

Three representatives of this new class of anti-diabetics are currently in the market-canagliflozin, dapagliflozin and empagliflozin. Some others are under development. Although agents differ in their affinity for the sodium-glucose transporter, their clinical efficacy is quite similar. All three have similar efficacy in terms of glucose control and all are associated with significant weight loss [106]. All three have been reported to significantly decrease systolic and diastolic BP by 3–5/2–3 mmHg [107]. A meta-analysis published in 2014 reported that SGLT2 inhibitors decrease systolic and diastolic BP by 4 and 1.6 mmHg compared with placebo [108]. A pooled analysis of studies of canagliflozin and dapagliflozin concluded that orthostatic hypotension was not increased during treatment with these SGLT2 inhibitors compared with placebo. Three independent studies published after this meta-analysis specifically evaluated the effect of SGLT2 inhibitors on BP in diabetic individuals. The dapagliflozin BP study [109] reported a 4.28 mmHg statistically significant decrease in seated systolic BP during a 12-week period compared with placebo and a non-statistically significant decrease in diastolic BP compared with placebo. Orthostatic hypotension was not increased in the treatment arm compared with placebo. In another study Weber MA et al. showed a 3.1 mmHg fall in seated systolic BP and 2.9 mmHg fall in systolic BP recorded by 24H ambulatory blood pressure monitoring after 12 weeks of treatment with dapagliflozin[110]. The Empagliflozin Cardiovascular Outcome Event Trial in Type 2 Diabetes Mellitus Patients (EMPA-REG) BP study [111] reported that mean systolic BP as evaluated by ambulatory BP monitoring was significantly lower in patients treated with both 10 and 25 mg empagliflozin compared with placebo. Patients who entered the study with uncontrolled hypertension had a more significant response to empagliflozin compared with those in which BP was well controlled prior to study initiation. Orthostatic hypotension was more prevalent in the empagliflozin group, but none of the patients who had orthostatic hypotension experienced clinical events related to this finding, making its clinical significance questionable. The canagliflozin BP study was the smallest of the three studies [112] and reported findings consistent with the previously mentioned studies with a decrease in systolic BP as assessed by ambulatory BP monitoring of approximately 2 mmHg for both 100 and 300 mg. Orthostatic hypertension as assessed by ambulatory BP monitoring or through clinical symptoms occurred only in the canagliflozin group. It is important to note that patients enrolled to all three studies of SGLT2 inhibitors in hypertensive diabetics were Caucasian and data evaluating the influence of SGLT inhibitors on BP in diabetics of non-Caucasian origin is much less extensive.

**Fig. 1:**
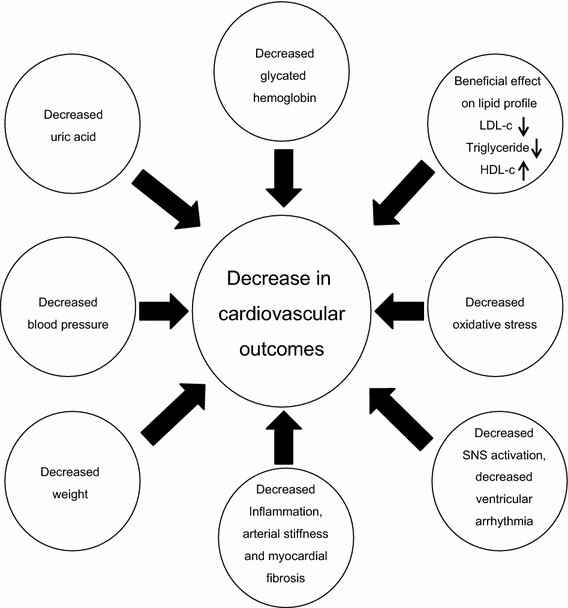
Beneficial effects of SGLT2 inhibitors, SNS, sympathetic nervous system; LDL-c, low density lipoprotein cholesterol; HDL, high density lipoprotein cholesterol

In addition, these drugs were reported to have a positive effect on the circadian rhythm in rats who developed hypertension [113]. The mechanism underlying the BP decrease by SGLT2 inhibitors is unclear and potential mechanisms include diuresis, nephron remodeling, decrease in arterial stiffness, and weight loss [114]. This class of agents is certainly promising as it can be used to control glucose, weight and BP. In fact, the EMPA-REG trial indeed showed that empagliflozin is associated with decreased CV morbidity, CV mortality and overall mortality [115]. Several potential non-glycemic mechanisms such as BP decrease and weight reduction have been suggested to explain the CV benefit of SGLT2 (Fig.  1). Whether SGLT2 inhibitors can be used for BP control in non-diabetic individuals is unclear. The results of the Canagliflozin Cardiovascular Assessment Study (CANVAS) [116] and DECLARE-TIMI 55 (for dapagliflozin) [117] studies which are expected to be complete on 2017 and 2019 respectively will clarify whether the CV benefits reported for empagliflozin are a class-effect.

## Conclusions

Current evidence does not support a more stringent BP control strategy for all diabetic patients and the evidence to support stringent control in certain diabetic patients is also inconclusive. In elderly diabetic patients (>80 years) BP levels should be less than 140–150/90 mmHg and should be monitored closely in the sitting and the standing position and the treatment should be tailored to prevent excessive fall/decrease in BP. This is reflected in recommendations in most current BP treatment guidelines. The choice of anti-hypertensive agent is supported by minimal evidence although RAAS blockers are usually used as first-line agents. When requiring more than one agent for the control of hypertension in diabetics, calcium antagonists or diuretics are probably appropriate as second line agents. New agents used for the treatment of diabetes may aid in the control of hypertension and a diagnosis of hypertension in a diabetic person may influence the clinician’s choice to use a certain anti-diabetic treatment.

In addition to lowering BP it is very important to control all other risk factors in diabetic patients. This heterogeneous treatment model, relates directly to general trends in modern medicine, reflecting an aspiration for individually tailored medicine, adapted specifically for the particular demographic and biologic characteristics of each patient.

## Abbreviations

DM: diabetes mellitus
BP: blood pressure
CV: cardiovascular
